# Safety, tolerability, and effectiveness of the sodium-glucose cotransporter 2 inhibitor (SGLT2i) dapagliflozin in combination with standard chemotherapy for patients with advanced, inoperable pancreatic adenocarcinoma: a phase 1b observational study

**DOI:** 10.1186/s40170-023-00306-2

**Published:** 2023-05-18

**Authors:** Lauren K. Park, Kian-Huat Lim, Jonas Volkman, Mina Abdiannia, Hannah Johnston, Zack Nigogosyan, Marilyn J. Siegel, Janet B. McGill, Alexis M. McKee, Maamoun Salam, Rong M. Zhang, Da Ma, Karteek Popuri, Vincent Tze Yang Chow, Mirza Faisal Beg, William G. Hawkins, Linda R. Peterson, Joseph E. Ippolito

**Affiliations:** 1 Department of Medicine, Cardiovascular Division, Washington University School of Medicine in St. Louis, Mail Stop Code: 8131, 660 S. Euclid Ave., Saint Louis, MO 63110 USA; 2 Department of Medicine, Oncology Division, Washington University School of Medicine in St. Louis, St. Louis, MO USA; 3 Mallinckrodt Institute of Radiology, Washington University School of Medicine in St. Louis, Mail Stop Code: 8131, 4559 Scott Ave., St. Louis, MO 63110 USA; 4 Department of Medicine, Division of Endocrinology, Metabolism and Lipid Research, Washington University School of Medicine in St. Louis, St. Louis, MO USA; 5 Department of Internal Medicine, Section of Gerontology and Geriatric Medicine, Wake Forest University School of Medicine, Winston-Salem, NC USA; 6 Department of Computer Science, Memorial University of Newfoundland, St. John’s, NL Canada; 7 School of Engineering Science, Simon Fraser University, Burnaby, BC Canada; 8 Department of Surgery, Washington University School of Medicine in St. Louis, St. Louis, MO USA; 9 Department of Biochemistry and Molecular Biophysics, Washington University School of Medicine in St. Louis, St. Louis, MO USA

**Keywords:** Pancreatic ductal adenocarcinoma, Safety, Efficacy, Sodium-glucose cotransporter-2 inhibitor, SGLT2, Dapagliflozin

## Abstract

**Background:**

Pancreatic ductal adenocarcinoma (PDAC) is a lethal malignancy. Thus, there is an urgent need for safe and effective novel therapies. PDAC’s excessive reliance on glucose metabolism for its metabolic needs provides a target for metabolic therapy. Preclinical PDAC models have demonstrated that targeting the sodium-glucose co-transporter-2 (SGLT2) with dapagliflozin may be a novel strategy. Whether dapagliflozin is safe and efficacious in humans with PDAC is unclear.

**Methods:**

We performed a phase 1b observational study (ClinicalTrials.gov ID NCT04542291; registered 09/09/2020) to test the safety and tolerability of dapagliflozin (5 mg p.o./day × 2 weeks escalated to 10 mg p.o./day × 6 weeks) added to standard Gemcitabine and nab-Paclitaxel (GnP) chemotherapy in patients with locally advanced and/or metastatic PDAC. Markers of efficacy including Response Evaluation Criteria in Solid Tumors (RECIST 1.1) response, CT-based volumetric body composition measurements, and plasma chemistries for measuring metabolism and tumor burden were also analyzed.

**Results:**

Of 23 patients who were screened, 15 enrolled. One expired (due to complications from underlying disease), 2 dropped out (did not tolerate GnP chemotherapy) during the first 4 weeks, and 12 completed. There were no unexpected or serious adverse events with dapagliflozin. One patient was told to discontinue dapagliflozin after 6 weeks due to elevated ketones, although there were no clinical signs of ketoacidosis. Dapagliflozin compliance was 99.4%. Plasma glucagon increased significantly. Although abdominal muscle and fat volumes decreased; increased muscle-to-fat ratio correlated with better therapeutic response. After 8 weeks of treatment in the study, partial response (PR) to therapy was seen in 2 patients, stable disease (SD) in 9 patients, and progressive disease (PD) in 1 patient. After dapagliflozin discontinuation (and chemotherapy continuation), an additional 7 patients developed the progressive disease in the subsequent scans measured by increased lesion size as well as the development of new lesions. Quantitative imaging assessment was supported by plasma CA19-9 tumor marker measurements.

**Conclusions:**

Dapagliflozin is well-tolerated and was associated with high compliance in patients with advanced, inoperable PDAC. Overall favorable changes in tumor response and plasma biomarkers suggest it may have efficacy against PDAC, warranting further investigation.

**Supplementary Information:**

The online version contains supplementary material available at 10.1186/s40170-023-00306-2.

## Background

Pancreatic ductal adenocarcinoma (PDAC) remains highly lethal with an overall 5-year survival rate of ~11%, and the survival rate for those with distant metastases is a dismal 3% [1, 2]. Combination chemotherapies such as Gemcitabine plus nab-paclitaxel (GnP) and FOLinic Acid, 5-Fluorouracil, IRINotecan, and OXaliplatin (FOLFIRINOX) remain the mainstay of treatment over the past decade [3, 4]. However, these regimens are associated with significant side effects and treatment responses are typically short-lived. Therefore, therapeutic strategies that can augment treatment response without incurring further toxicities are an urgent, unmet clinical need.

Deregulation of cellular metabolism is an established hallmark of tumorigenesis and a clinically relevant avenue for new therapeutics [5]. In fact, several lines of evidence have demonstrated the prognostic potential of glucose utilization in pancreatic cancer. Fluorodeoxyglucose positron emission tomographic (FDG-PET) imaging has been shown to be a highly sensitive modality for pancreatic cancer detection with a pooled sensitivity of 90% [6, 7]. Enhanced glycolysis has also been recognized as an important metabolic alteration in PDAC metastasis that involves the epithelial–mesenchymal transition, angiogenesis, and subsequent seeding of cancer cells in distant organs [8, 9]. One mechanism for this phenomenon is the conversion of glucose into energy, anabolic pathways, and reducing equivalents to mitigate oxidative stress [10, 11]. This has clinical significance, as the presence of diabetes and increased circulating glucose increases the risk of PDAC [12]. Thus, novel anti-diabetic interventions that inhibit tumor glucose uptake or metabolism may have efficacy in patients with PDAC.

Sodium-glucose co-transporter 2 inhibitors (SGLT2i) are a new, FDA-approved class of glucose-lowering drugs that inhibit renal glucose reabsorption and reduce plasma glucose through increased urinary glucose excretion. SGLT2i improves all-cause survival and decreases major cardiovascular events in patients with type 2 diabetes and in those with heart failure [13, 14]. Although SGLT2 protein (from *SLC5A2*) is expressed primarily in normal kidneys, recent data show that several human tumors upregulate SGLT2, including PDAC, brain, lung, colon, and prostate [15–19]. In fact, functional SGLT2 protein is expressed in human PDAC tumors. Moreover, SGLT2 has been recently shown to promote PDAC progression by activation of Hippo signaling via the hnRNPK-YAP1 axis [20]. Inhibition of SGLT2 in preclinical PDAC models results in tumor necrosis and decreased growth [17]. Not only do SGLT2 inhibitors (SGLT2i) have direct anti-tumor effects, but this class of drugs has beneficial pleiotropic effects including reversing hyperinsulinemia [21, 22], decreasing plasma glucose, producing a mild ketonemia, and reducing visceral obesity [23–30], all of which can improve survival in patients with cancer, particularly PDAC [31–35].

We conducted a phase 1b clinical trial combining the SGLT2i dapagliflozin with standard GnP chemotherapy for patients with newly diagnosed, advanced PDAC. We chose dapagliflozin because of its efficacy in xenograft models of PDAC [17] and our team’s extensive experience administering dapagliflozin.

## Methods

### Study design and participants

This study was approved by the Washington University in St. Louis Institutional Review Board (HRPO#202011019) and registered as NCT04542291 on September 09, 2020. The study was conducted in accordance with the *Declaration of Helsinki*. Inclusion criteria included histologically or cytologically confirmed metastatic or locally advanced pancreatic ductal adenocarcinoma or pancreatic adenosquamous carcinoma, ≥ 18 years of age, no prior systemic treatment, preserved organ and hematologic functions, lack of brain metastases, Eastern Cooperative Oncology Group performance status (ECOG PS) 0 or 1, and in which GnP therapy was deemed appropriate by the treating oncologists. Exclusion criteria included current or previous treatment with an SGLT2i, thiazolidinedione, or insulin use for diabetes at baseline, chronic steroid use, baseline HbA1c > 10%, history of stroke or transient ischemic attack in preceding 5 years, and uncontrolled intercurrent illnesses. All subjects provided written informed consent.

### Procedures

Following enrollment, patients were treated with Gemcitabine and nab-Paclitaxel starting from 1000 mg/m^2^ and 125 mg/m^2^, respectively, on days 1, 8, and 15 of every 28-day cycle. Dapagliflozin treatment was initiated at 5 mg once daily for the first 2 weeks and escalated to 10 mg once daily after assessment by an endocrinologist for an additional 6 weeks, totaling 8 weeks or 2 cycles of GnP. If a patient was deemed to have a progression of pancreatic insulin insufficiency and/or significantly increasing blood glucose levels during the study, the treating endocrinologist was allowed to add other glucose-lowering medications to the study drug (dapagliflozin) for clinical purposes. Patients were seen in the clinic or followed up via phone visits every 3–4 days and weekly for a check on ketone status and side effects according to medical need. Repeat imaging studies were performed after 8 weeks of treatment, and the overall response was classified as stable disease (SD), partial response (PR), or progressive disease (PD) using Response Evaluation Criteria in Solid Tumors (RECIST 1.1; discussed below) [36, 37]. Patients stayed on GnP alone until PD occurred or drug intolerance developed. Patients were evaluated for toxicity and adverse events for 3 months after discontinuation of dapagliflozin. In addition, patients were instructed to measure urine ketones routinely on a weekly basis or when feeling sick (Ketodiastix; Bayer, Germany) to monitor for ketoacidosis.

### Outcomes measurements

#### Adverse events

Treatment-related adverse events were documented during every clinic visit and the severity scored using the Common Terminology Criteria for Adverse Events (CTCAE) v.5 classifications (Grade 1: mild; Grade 2: moderate; Grade 3: severe; Grade 4: life-threatening requiring urgent interventions; Grade 5: death).

#### Laboratory value analysis

The following blood laboratory values were assessed prior to and at the end of study treatment (EOT; 8 weeks): fasting glucose, c-peptide (a biomarker for endogenous insulin production), glucagon, beta-hydroxybutyrate ketone, and pancreatic polypeptide (a secreted biomarker for the endocrine pancreas). The tumor marker CA19-9 was monitored during treatment, along with a complete blood count (CBC) and comprehensive metabolic panel (CMP). Any value reported as less than the threshold (e.g., < 0.2 mmol/L) was treated as below the detection limit and not plotted in the analyses. Percent change of values at EOT relative to baseline was calculated as (− 1) × [1-(EOT value/baseline value)] × 100%.

#### Tumor imaging/quantification analysis

CT examinations in Digital Imaging and Communications in Medicine (DICOM) format at baseline and EOT were transferred to a workstation equipped with Mint Lesion software (Mint Medical; Hamilton, NJ). Tumor measurements were performed by a single radiologist using RECIST 1.1 criteria, and an overall response assessment was generated [36, 37]. Both target and non-target lesions were assessed. Percent change in the sum of the diameters (SODs) of the target lesions relative to baseline was calculated as (− 1) × [1-(EOT value/baseline value)] × 100%. Although baseline and EOT imaging were the primary emphases for this trial, CT scans performed following the completion of the study (when available) as part of the standard of care were also analyzed to measure tumor response.

#### CT-based quantitative body composition assessment

DICOM-formatted abdominal CT examinations that were used for RECIST measurements at baseline and EOT were obtained. Scans were transferred to a workstation equipped with the data analysis facilitation suite (DAFS; Voronoi Health Analytics; British Columbia, Canada). This software platform has been previously validated to assess the effects of body composition on the mortality of patients with cancer [38–41]. Automated segmentation of the fat and muscle was performed by the software and verified by a board-certified abdominal radiologist (JEI). Volumetric analyses were performed from the L1–L4 levels, as these levels were present in their entirety in a single continuous scan for all patients at both baseline and EOT.

### Statistical analysis

Continuous variables were reported as means (± standard deviation), and categorical variables were reported as proportions, unless otherwise specified. Comparisons of measurements between baseline and EOT were performed by using the Student’s paired *t* test. Correlations were assessed by using the Pearson correlation coefficient (r). Statistical analyses were performed by using Prism 9.1.0 (GraphPad Software, La Jolla, CA, USA). Two-tailed statistical tests were performed where applicable, and *p* < 0.05 was considered to indicate a statistically significant difference.

## Results

### Subject characteristics

**Fig. 1:**
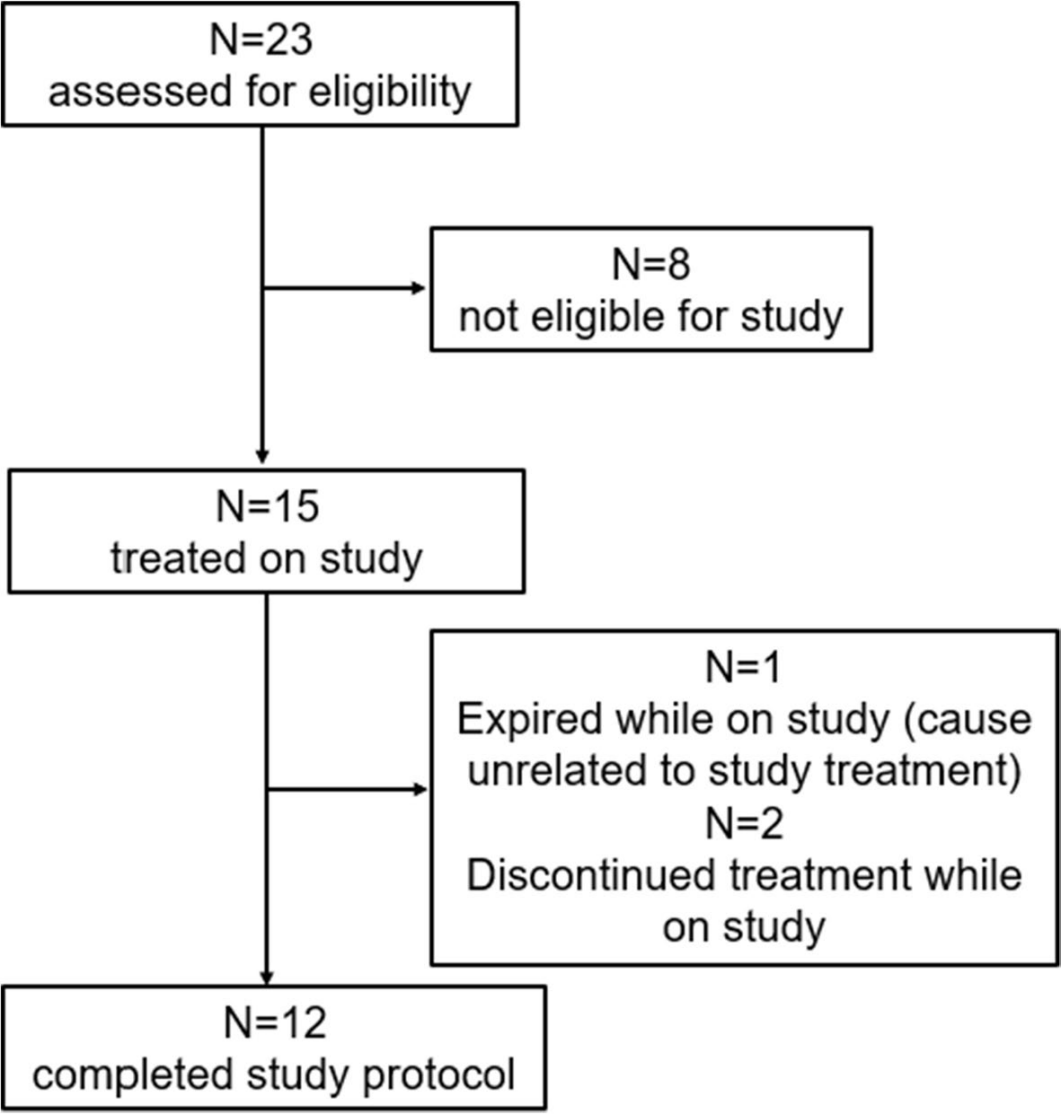
Consort diagram displaying the progression of patients through the study protocol from initial assessment for eligibility to protocol completion

Between March and September 2021, 23 patients with newly diagnosed locally advanced or metastatic PDAC were screened, and 15 patients were enrolled (Fig. 1). Of these 15 patients, three patients dropped out during the first 4 weeks of treatment start. One patient developed severe upper gastrointestinal hemorrhage secondary to primary tumor invasion and after clinical stabilization decided to pursue hospice. Two patients elected to withdraw from the study due to intolerance to GnP chemotherapy as determined by the treating oncologists and were treated with a reduced dose of GnP or single-agent gemcitabine. The baseline characteristics of the remaining 12 evaluable patients are shown in Table 1. Ten patients had ductal adenocarcinoma and two had a mixed adenosquamous (> 50% adenocarcinoma) histology. The average age was 66.8 years, and 92% of patients were white. The baseline plasma CA19-9 was elevated in nine of twelve evaluable patients. Only one patient had a history of type 2 diabetes at the time of entry into the study, but two other patients met the criteria for diabetes by either a fasting glucose concentration > 126 mg/dL and/or a hemoglobin A1c > 6.5% during the study. None was taking any glucose-lowering medications at baseline (Supplementary Table 1). However, during the study, the two who developed diabetes despite dapagliflozin treatment were started on glucose-lowering therapy (one on basal insulin and one on metformin). The most common medications taken by the patients in addition to dapagliflozin and GnP included acetaminophen (92%), opiates (67%), and antiemetics (42%).

**Table 1:** Baseline characterisstics of evaluable study participants

	***N* (frequency %) or mean [SD]**
Age (years)	66.75 [5.1]
Sex	
Male	5 (42%)
Female	7 (58%)
Race	
White	11 (92%)
Black	1 (8%)
Height (cm)	170.4 [7.8]
Weight (kg)	81.4 [11.8]
Body mass index (kg/m^2^)	27.9 [4.2]
HbA1c (%)	5.9 [0.9]
***Histology***	
Pancreatic ductal adenocarcinoma	10 (83%)
Pancreatic adenosquamous carcinoma	2 (17%)
***Extent of disease***	
Metastatic	10 (83%)
Locally advanced	2 (17%)
***Pretreatment labs***	
Albumin (> 3.5 g/dL)	11 (92%)
CA19-9 (> 35 U/mL)	10 (83%)
Sodium (mmol/L)	138.8 [2.7]
Potassium (mmol/L)	3.9 [0.4]
Creatinine (mg/dL)	0.6 [0.1]
Calcium (mg/dL)	9.1 [0.7]
Glucose (mg/dL)	108.8 [25.1]

Data are presented as *N* (frequency %) or mean (SD)

*SD* Standard deviation, *HbA1c* Hemoglobin A1c

#### Compliance, safety, and tolerability

Eleven of the 12 evaluable patients completed 2 weeks of 5 mg/day of dapagliflozin followed by 6 weeks of 10 mg/day as planned. One patient had 2+ urinary ketones on urinalysis, albeit without clinical ketoacidosis or increased anion gap, and was instructed to discontinue dapagliflozin 2 weeks before the end of the trial. He completed the post-study imaging and blood sampling. All other patients completed 8 weeks of dapagliflozin without deviation. Prior to his discontinuation, he had been 100% compliant. Dapagliflozin compliance for all other patients enrolled (*N* = 14) was 99.4%. Side effects were attributed to dapagliflozin, GnP, or both by the treating oncologists for each patient and presented in Table 2. There were no grade 3–4 side effects attributed to dapagliflozin alone. Side effects attributed to dapagliflozin were infrequent and mild (grades 1–2) and included fatigue, nausea, dry mouth, and plasma sodium levels. The majority of side effects attributed to the study regimen overall were related to the known side effects of GnP (Table 2). The treating physicians in charge of monitoring side effects concluded that the addition of dapagliflozin to GnP was generally well-tolerated and did not inflict additional toxicities on almost all participants in this study.

**Table 2:** Adverse event profiles by CTCAE v5.0 of all 15 enrolled patients during the trial

	**Related to dapagliflozin only**		**Related to GnP**		**Related to both dapagliflozin and GnP**		**Related to all**	
	**Grade 3/4**	**All grades**	**Grade 3/4**	**All grades**	**Grade 3/4**	**All grades**	**Grade 3/4**	**All grades**
**Constitutional**								
Anorexia	0	0	0	4 (27%)	0	1 (7%)	0	5 (33%)
Headache	0	0	0	1 (7%)	0	0	0	1 (7%)
Fatigue	0	1 (7%)	0	3 (20%)	0	2 (13%)	0	6 (40%)
Nausea	0	1 (7%)	0	1 (7%)	0	4 (27%)	0	6 (40%)
Vomiting	0	0	0	0	0	3 (20%)	0	3 (20%)
**Hematologic**								
Leukopenia	0	0	1 (7%)	7 (47%)	1 (7%)	3 (20%)	2 (13%)	10 (67%)
Neutropenia	0	0	1 (7%)	5 (33%)	1 (7%)	2 (13%)	2 (13%)	7 (47%)
Lymphopenia	0	0	1 (7%)	5 (33%)	0	2 (13%)	1 (7%)	7 (47%)
Anemia	0	0	0	9 (60%)	0	6 (40%)	0	15 (100%)
Thrombocytopenia	0	0	0	7 (47%)	0	5 (33%)	0	12 (80%)
Neutropenic fever	0	0	0	0	1 (7%)	1 (7%)	1 (7%)	1 (7%)
**Gastro-intestinal tract**								
Dry mouth	0	1 (7%)	0	0	0	1 (7%)	0	2 (13%)
Constipation	0	0	0	1 (7%)	0	0	0	1 (7%)
Diarrhea	0	0	1 (7%)	4 (27%)	1 (7%)	2 (13%)	2 (13%)	6 (40%)
Colitis	0	0	0	1 (7%)	0	0	0	1 (7%)
**Liver**								
Hypoalbuminemia	0	0	0	0	0	2 (13%)	0	2 (13%)
Increased ALT	0	0	0	4 (27%)	0	5 (33%)	0	9 (60%)
Increased AST	0	0	0	2 (13%)	0	5 (33%)	0	7 (47%)
Increased ALP	0	0	0	2 (13%)	0	4 (27%)	0	6 (40%)
**Kidney and electrolytes**								
Hyponatremia	0	1 (7%)	0	2 (13%)	0	0	0	3 (20%)
Proteinuria	0	0	0	0	0	2 (13%)	0	2 (13%)
Elevated creatinine	0	0	0	0	0	2 (13%)	0	2 (13%)
**Dermatologic**								
Alopecia	0	0	0	10 (67%)	0	0	0	10 (67%)
Rash	0	1 (7%)	0	1 (7%)	0	1 (7%)	0	3 (20%)
**Nervous system**								
Peripheral sensory neuropathy	0	0	0	4 (27%)	0	0	0	4 (27%)
Dysgeusia	0	0	0	1 (7%)	0	0	0	1 (7%)

#### Metabolic response

**Fig. 2:**
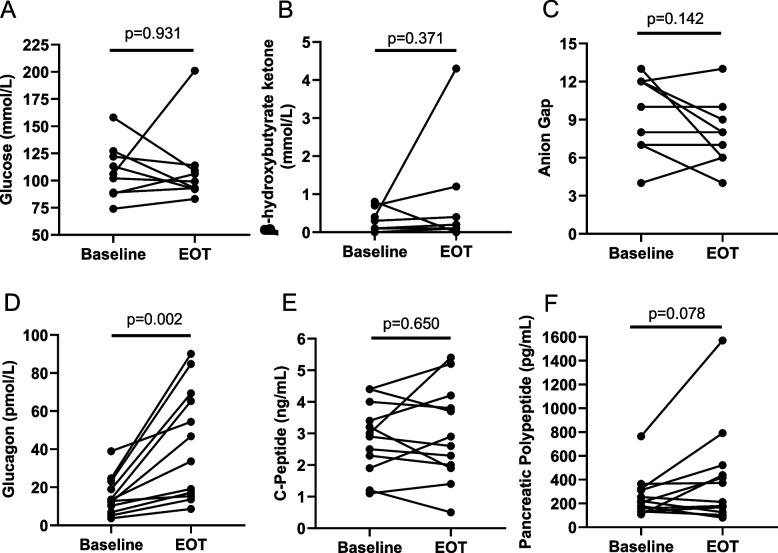
Changes in plasma metabolic chemistry following dapagliflozin treatment. Fasting metabolic lab values were obtained of **A** glucose, **B** b-hydroxybutyrate ketone, **C** anion gap, an indirect measurement of plasma ketoacids, **D** glucagon, **E** C-peptide (a marker for endogenous insulin), and **F** and pancreatic polypeptide were measured at the first day of treatment (day 1) and the 15th day of the second cycle of treatment (day 43). Paired *t* tests across patients showed only a significant increase in glucagon

Metabolic responses (assessed by plasma chemistries) of the 12 patients who completed the protocol were measured at baseline before initiation of dapagliflozin and at EOT. There was no significant change in plasma glucose or beta-hydroxybutyrate levels (Fig. 2A, B). The lack of significant change in beta-hydroxybutyrate is supported by the concomitant lack of change in the anion gap, an indirect measure of circulating ketoacids (Fig. 2C). However, plasma glucagon levels significantly increased (average 2.8-fold increase; Fig. 2D). In contrast, plasma c-peptide levels (a marker for endogenous insulin production) were not significant (Fig. 2D). Change in pancreatic polypeptide (a marker of pancreatic endocrine and exocrine function) concentrations [42] trended toward significance (*p* = 0.078) with an average 1.6-fold increase at EOT (Fig. 2E).

#### Tumor response

**Fig. 3:**
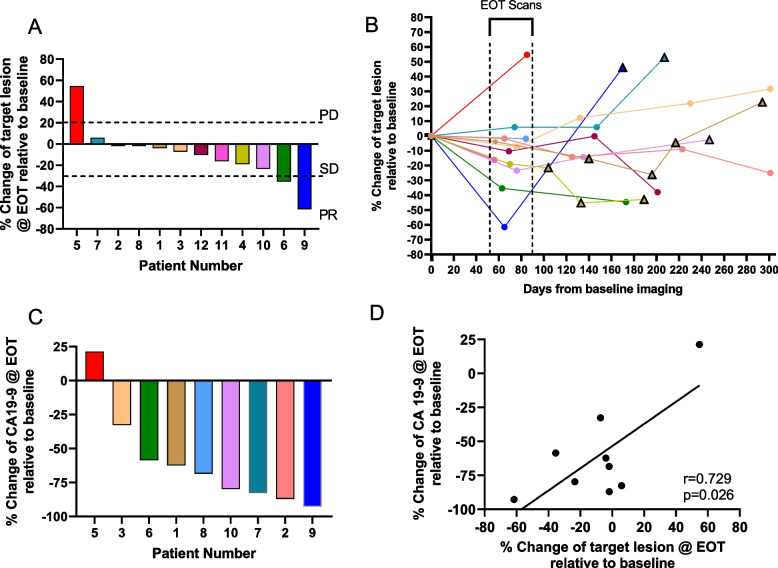
Changes in tumor response over dapagliflozin treatment. **A** Waterfall plot of percent change of RECIST 1.1 target measurements at end of treatment (EOT) using baseline as a reference identify 1 patient with progressive disease (PD), 9 patients with stable disease (SD), and 2 patients with partial response (PR). **B** CT scans for RECIST 1.1 target measurements were available for 11 of the 12 patients following EOT, identifying that the majority of patients (8/11) either developed new lesions at multiple timepoints (triangles) or experienced a > 30% increase in target lesion size. **C** With the exception of 1 patient, CA 19-9 decreased in all patients. CA19-9 was not detectable in patients 4 and 12 and not obtained at EOT for patient 11. **D** A positive correlation exists between CA 19-9 tumor marker expression and RECIST-based tumor measurements. Patients are uniformly color-coded across all panels

The response to therapy was assessed using RECIST 1.1 criteria as well as plasma CA19-9 levels, a clinical biomarker used for PDAC. Of the 12 evaluable patients, after the first 8 weeks/2 cycles of treatment, two had a partial response (PR), defined as greater than 30% reduction in target lesion size. The majority (9/12) had stable disease (SD), and only one patient had progressive disease (PD), defined as a greater than 20% increase in target lesion size (Fig. 3A). RECIST analysis was expanded for these patients outside of the dapagliflozin treatment window when they were only receiving GnP. After dapagliflozin treatment ended, with subsequent imaging, the majority (7/11) of patients experienced progression of the disease characterized by either (i) the presence of new metastatic lesions or (ii) the increase in target lesion size greater than 30% relative to baseline (Fig. 3B), despite continuing on GnP therapy.

These imaging data were compared to plasma CA19-9 levels. Three of the twelve patients had normal CA19-9 levels at diagnosis and hence not included. For the remaining 9 patients, the percent change of CA19-9 largely mirrored the changes in tumor size as measured using RECIST. Only one patient experienced an increase (~ 50%) in tumor biomarker burden, as well as tumor size (Fig. 3A–D). Moreover, there was a significant positive correlation (*r* = 0.729; *p* = 0.026) between the percent change in RECIST tumor size and the percent change in CA19-9 (Fig. 3D).

#### Change in body composition

**Fig. 4:**
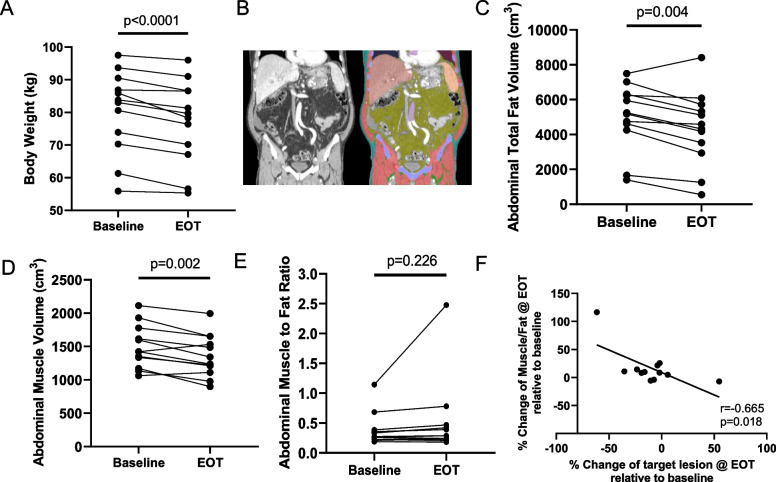
Changes in body composition following dapagliflozin treatment. **A** Body weight significantly decreases during treatment. **B** Automated segmentation of CT datasets from study patients was performed to quantify the total abdominal fat and muscle. **C**, **D** Total abdominal fat and muscle volume decrease during treatment. **E** However, the muscle-to-fat ratio increased robustly in the extreme responder in the trial. **F** There is a significant positive correlation in the % change in the muscle-to-fat ratio versus % change in RECIST tumor target measurement, suggesting that maintaining muscle mass while losing fat is important for robust therapeutic response

Because SGLT2i therapy as well as pancreatic cancer are known to alter fat and muscle mass, [27, 43, 44], volumetric abdominal muscle and fat measurements were obtained from CT scans on the patients performed at baseline and at EOT. First, every patient lost weight during the course of treatment with GnP + dapagliflozin (Fig. 4A). A validated automated body composition algorithm was used to compute changes in total abdominal skeletal muscle and fat volumes (Fig. 4B) [42]. On average, both fat and muscle mass decreased in these patients (Fig. 4D, E). However, when the muscle-to-fat ratio was computed for these patients, no overall significant differences were identified. One patient had a greater than twofold increase in the muscle-to-fat ratio over the course of dapagliflozin therapy (Fig. 4E). This patient was also the patient that had the best RECIST response (Fig. 3A, B). To evaluate this effect further, a correlation was performed on the change in the muscle-to-fat ratio versus the change in the RECIST quantitative measurements (SOD). A modest but significant correlation existed between muscle-to-fat ratio and RECIST measurements. Patients who maintained more muscle mass and lost more fat mass during dapagliflozin therapy had better quantitative RECIST responses than those who did not (Fig. 4F).

## Discussion

To our knowledge, this is the first prospective study in humans to evaluate the safety and tolerability of the SGLT2i, dapagliflozin, as adjunctive therapy in combination with chemotherapy for patients with PDAC. It is also the first to preliminarily explore the potential efficacy of dapagliflozin on PDAC. This phase 1b study demonstrated that dapagliflozin, added to standard GnP chemotherapy, was very well-tolerated and associated with few adverse symptoms. Moreover, patients were highly compliant with dapagliflozin. There was also evidence suggesting that dapagliflozin, in addition to GnP, was associated with salutary effects on PDAC. Stable disease and partial response were maintained in 11 of 12 patients that were accompanied by a decrease in CA19-9. In addition, quantitative changes in body composition were identified where maintenance of muscle mass and loss of fat mass were associated with better quantitative tumor response measurements. In sum, there is evidence that dapagliflozin may potentially be efficacious as adjunctive therapy for advanced PDAC treatment, and dapagliflozin is generally very well-tolerated and generally safe to use in this very ill population.

Before this trial, it was not clear that SGLT2i therapy would be generally well-tolerated by this population of patients with advanced, inoperable PDAC because they are often symptomatic and have an average survival of ~12 months [3, 4]. Patients taking any SGLT2i therapy need to stay hydrated and take in adequate amounts of carbohydrates to avoid ketoacidosis, and patients with advanced PDAC often experience nausea and vomiting as hallmark manifestations of their disease and/or treatment with GnP chemotherapy. Knowing this, patients in this trial were instructed on recommended carbohydrate intake, encouraged to stay hydrated, and were instructed to measure their ketone levels frequently. They were also followed closely by our interdisciplinary study team, including board-certified endocrinologists. Likely in part due to this proactive protocol, the vast majority of patients were able to complete the study and avoid ketoacidosis. The one patient who was told near the end of the study to discontinue dapagliflozin due to 2+ urine ketone levels was likely dehydrated, as evidenced by a urine specific gravity > 1.03, due to episodes of nausea and vomiting. However, the overall treated-related adverse event profile, as shown in Table 2, was very favorable for dapagliflozin, and most adverse events and all grade 3–4 adverse events during the trial were attributed to GnP. The low percentage of side effects seen in the current study is similar to that seen in other larger multicenter controlled studies, such as the DECLARE-TIMI 58 trial [14]. In that study of patients who had type 2 diabetes and cardiovascular disease or who were at increased risk for cardiovascular disease, most side effects occurred in 1% or less of the patients studied. Of note, no patient had a significantly elevated creatinine that was attributed to dapagliflozin. This is consistent with the known small decrease in glomerular filtration rate that can occur in shorter-term interventions (after ~2–4 weeks, likely due to a decrease in intraglomerular pressure) which typically recovers at least in part in subsequent weeks and is often followed by preservation of renal function in the long term [45, 46]. In short, dapagliflozin was well-tolerated by almost all patients with PDAC in the current trial, which likely contributed to the extremely high drug compliance rate in our study.

Dapagliflozin was not only well-tolerated in the current trial, but there were also potential signs of its efficacy as a treatment for PDAC when added to standard GnP therapy. Eleven of twelve evaluable patients had either stable disease or partial response during the initial 8 weeks of study treatment. Plasma CA19-9 levels showed similar trends. Perhaps most indicative of a therapeutic effect of the dapagliflozin, several patients had an increase in tumor size or developed new lesions after the dapagliflozin was stopped, even though the patients were continued on standard-of-care chemotherapy. These data suggest that direct metabolic modulation with SGLT2i is not only safe and generally well-tolerated but also potentially an efficacious treatment versus PDAC. To give context to our findings, a phase 3 randomized clinical trial assessed the efficacy of GnP in patients with metastatic PDAC [4]. The rate of disease control in patients on GnP alone for 16 weeks or more was 48%. Our disease control rate over 8 weeks was 11/12 (91%). Though our study and the randomized trial are not directly comparable because patients were not followed for the same length of time and our study was a first-in-human, smaller study, our response rate is encouraging. Moreover, suggestive of a therapeutic effect of the dapagliflozin in our study, several patients had an increase in tumor size or developed new lesions after the dapagliflozin was stopped, even though the patients were continued on standard-of-care GnP. Although caution is recommended in extrapolating these results to dapagliflozin efficacy because of its limited duration in this study, these data indicate that direct metabolic modulation with SGLT2i is safe and generally well-tolerated and support future phase II studies combining SGLT2i with GnP until disease progression to better assesses the efficacy of this strategy.

Systemic metabolic effects were also seen after treatment with SGLT2i therapy in these patients with PDAC. Though at first glance it may be surprising that there was no change in fasting plasma glucose levels (Fig. 2A), none of the patients had uncontrolled diabetes or required insulin at baseline per the study protocol and the majority of patients (*N* = 9/12) did not have diabetes, so the average baseline fasting glucose level was within normal limits < 110 mg/dL. Thus, there was relatively little room for change in systemic glucose levels in these patients. Similarly, there were no significant increases in plasma ketones on average (It is interesting to note that the patient with the greatest response to dapagliflozin in terms of tumor regression had the greatest increase in plasma ketones). However, the lack of significant overall change in plasma glucose or ketone levels suggests that direct inhibition of glucose uptake through SGLT2 expression on PDAC [17] may play a more important anti-tumor role than indirect systemic changes in glucose. Though measurement of SGLT2-mediated glucose uptake in vivo with [^18^F]Me-4FDG positron emission tomography [17, 19] or SGLT2 expression in biopsy tissues pre- and post-dapagliflozin treatment were beyond the scope of this phase 1b trial, it would surely add to future studies of SGLT2i for human PDAC.

Although plasma ketone and glucose levels did not change overall, other systemic metabolic effects were noted with dapagliflozin treatment. Plasma glucagon levels increased in all subjects. This is a well-described effect of SGLT2i therapy and may suggest reduced beta-cell insulin secretion [47]. Supporting this notion, there was also a borderline increase in pancreatic polypeptide levels though C-peptide concentrations did not change. These systemic effects are very relevant for cancer therapy, as glucagon can have anti-tumor effects through unknown mechanisms [48, 49]. Though the primary aim of the current study was not to evaluate the change in pancreatic function, the increase in glucagon and borderline increase in pancreatic polypeptide levels suggest there may be an improvement in pancreatic function, as has been described in other studies of SGLT2i treatment in diabetes [50].

SGLT2i are known to decrease abdominal fat by increasing fatty acid oxidation [27, 44], which could be a reason why patients lost abdominal fat during the trial. However, this change can be confounded by the systemic metabolic effects of PDAC and chemotherapy that are also known to decrease body fat, mediated by anorexia, malabsorption, and/or tumor metabolic effects [51]. Paralleling the loss of abdominal fat, abdominal muscle mass also decreased during the trial. There has been some concern that the negative energy balance from SGLT2i therapy can result in loss of muscle mass as well as fat; free amino acids are released and transported to the liver for gluconeogenesis. Although this has been observed with other SGLT2i, this has not yet been documented with dapagliflozin [52]. Interestingly, although there were no significant effects on the abdominal muscle/fat ratio, patients who had a higher ratio at the end of therapy (i.e., retained more muscle mass and burned more fat) had better tumor response. This is supported by a multitude of data indicating that sarcopenia (i.e., low muscle mass), in addition to sarcopenic obesity (i.e., low muscle mass and high adiposity) are associated with poor outcomes in PDAC as well as multiple additional malignancies [31, 53–56]. In fact, our data are similar to a study by Babic et al. that examined the effects of postdiagnosis loss of skeletal muscle on outcomes in patients with advanced PDAC [57]. We observed an overall average 9% decrease in the skeletal muscle mass (over 8 weeks) which is strikingly similar to the ~ 10% decrease (over 8–16 weeks) observed by Babic et al., who demonstrated a correlation between preserved skeletal muscle mass and increased survival. Further, our findings relating to fat loss (− 18%) were also similar to those of Babic et al. (− 15%). These observations would be in line with our findings that less muscle mass loss during treatment correlated with greater response to therapy. Together, these data suggest that (i) metabolically targeted therapies that facilitate fat loss but maintain muscle mass may have a clinically significant impact on better PDAC patient outcomes and (ii) quantitative body composition assessment with CT can be used to potentially assess treatment response and predict better outcomes.

There are limitations to the current study. It is a small study by design as its primary aim was to evaluate safety and tolerability in patients with advanced PDAC. The size of the study necessarily limits the conclusions that can be drawn from it. Our study findings cannot be extrapolated to patients who did not fit our inclusion/exclusion criteria (e.g., who received different treatment options for PDAC or patients with other malignancies). Our study was not randomized or placebo-controlled, which would add to the study’s rigor. However, it did provide reassuring safety findings and intriguing efficacy signals.

In sum, our study findings demonstrate that dapagliflozin is generally well-tolerated and safe to use even in patients with advanced, inoperable PDAC. The favorable tolerability was accompanied by an extremely high compliance rate (> 99%). The study also suggested that dapagliflozin may have salutary effects on advanced PDAC when added as adjunctive therapy to standard GnP chemotherapy. The clinical implications of a new well-tolerated, oral medication which is potentially efficacious adjunctive therapy against metastatic PDAC are significant. A future larger randomized clinical trial is warranted to further evaluate the potential efficacy of SGLT2 inhibitor therapy in patients with PDAC.

## Supplementary Information

**Additional file 1: Supplementary Table 1.** Baseline Medication Use.

## Data Availability

The datasets used and/or analyzed during the current study are available from the corresponding author on reasonable request.
