# Supplementary material for: Safety, tolerability, and effectiveness of the sodium-glucose cotransporter 2 inhibitor (SGLT2i) dapagliflozin in combination with standard chemotherapy for patients with advanced, inoperable pancreatic adenocarcinoma: a phase 1b observational study
Source: Cancer Metab. 2023 May 18;11:6. doi: 10.1186/s40170-023-00306-2 (PMC10193807; doi:10.1186/s40170-023-00306-2)
Supplement: Supplementary file 1 — Additional file 1: Supplementary Table 1. Baseline Medication Use. [file 40170_2023_306_MOESM1_ESM.docx]

**Supplementary Table 1.** Baseline Medication Use (n=12)

| **Medication type** | Baseline |
| --- | --- |
|  | **n (%)** |
| Ace inhibitor/angiotensin receptor blocker | 4 (33) |
| Acetaminophen | 11 (92) |
| Anti-anxiolytic | 4 (33) |
| Antiemetic | 5 (42) |
| Aspirin | 2 (17) |
| Corticosteroid | 1 (8) |
| Opiates | 8 (67) |
| Proton Pump Inhibitors | 2 (17) |
| Thyroid replacement | 2 (17) |

*Note, one patient was started on long-acting insulin, and one was started on metformin during the study in addition to the study drug dapagliflozin for better glucose control.
